# The Nine Cancer Frames: A Tool to Facilitate Critical Reading of Cancer-Related Information

**DOI:** 10.1007/s13187-021-02062-7

**Published:** 2021-07-19

**Authors:** Craig Murray, Nina von Possel, Hanne C. Lie, Jarle Breivik

**Affiliations:** 1 Department of Behavioural Medicine, Institute of Basic Medical Sciences, Faculty of Medicine, University of Oslo, Blindern, P.O.Box 1111, N-0317 Oslo, Norway; 2 National Resource Centre for Late Effects After Cancer Treatment, Oslo University Hospital, Oslo, Norway

**Keywords:** Cancer communication, Framing analysis, Health literacy, Mass media, Social science, Medicine, UK, Norway

## Abstract

People’s ability to critically assess cancer-related information is essential from a preventional and therapeutic, as well as a general democratic perspective. Such cancer literacy is not just about acquiring factual knowledge. It also involves the ability to analyze how the information is contextualized—how cancer is framed. Previous research concerning the framing of cancer in public discourse is voluminous and penetrating but also fragmented and inaccessible to non-experts. In this study, we have developed an integrated and applicable tool for analyzing cancer discourse by systematically classifying distinctive ways of framing of the concept of cancer. Building on previous research and an inductive framing analysis of a broad range of public cancer discourse, systematically selected from British and Norwegian newspapers, we have characterized nine cancer frames: the biomedical, the environmental, the epidemiological, the personal, the sociopolitical, the economic, the antagonistic, the alternative, and the symbolic frame. This framing scheme may be applied to analyze cancer-related discourse across a plurality of themes and contexts. We also show how different frames combine to produce more complex messages, thereby revealing underlying patterns, strategies, and conflicts in cancer communication. In conclusion, this analytical tool enables critical reading of cancer-related information and may be especially useful in educational initiatives to advance health communication and public understanding of cancer.

## Introduction

Cancer literacy, meaning people’s ability to critically assess cancer-related information, is increasingly important. Overall cancer incidence and prevalence are rising, primarily because of an aging population [1]. The worldwide annual cost of cancer is estimated to more than US$ 1 trillion. Cancer therapy is a booming industry, and cancer research is a major driver of the ongoing biotechnological revolution [2]. To assure democratic decision-making and development, there is thus a strong need for the general population to understand cancer and its impact on society.

At the individual level, the word cancer is laden with fear and stigma [3]. Cancer prevention, screening, and therapy involve difficult questions related to ethics and equality [4, 5], and people’s understanding of these issues has immediate effects on health awareness, care-seeking behavior, and engagement in screening programs [6, 7]. How patients understand cancer influences how they relate to the disease and potentially its course of development, their quality of life, and even survival [8, 9].

Overall, there is a strong need for tools and methods that facilitate the development of cancer literacy. People should be able to critically assess cancer-related information from difference sources, especially the wide range of content provided by the mass media. Besides formal education, the media represent the public’s primary source of information regarding health and science [10, 11]. TV and online news channels convey the voices of patients, researchers, healthcare providers, and public institutions, and the media both shape and reflect public understanding of cancer.

Developing cancer literacy is not just a matter of acquiring factual knowledge about the relevant issues. It also involves the ability to analyze how the information is contextualized. To make sense of cancer—or any other issue—information has to be organized into an intellectual framework; it has to be *framed* [12, 13]. When people communicate, they frame their stories by using certain words, facts, depictions, metaphors, sources of information, and images. Accordingly, a particular concept may be presented or described in ways that communicate different meanings. A gene, for example, may be framed as a physical entity, a risk factor, or a symbol of relationship, depending on the context and the purpose of the communication [14]. Each frame conveys a different interpretation of reality [15], and framing analysis is a powerful tool, in research as well as education, for exploring the underlying process of communication [16].

Many studies have investigated the framing of cancer-related discourse. Some have focused on a single issue and identified narrow, *issue-specific* frames. Kolker [17], for example, found that patient advocacy groups frame breast cancer as an *epidemic*, a *problem of gender equality*, or a *threat to families*. Others have identified more generic frames, including *conflict*, *human impact*, and *economic consequences*, or *episodic* versus *thematic* news frames. Andsager and Powers categorized breast cancer coverage into a *basic information*, a *research*, and a *personal stories* frame [18], whereas Park and Reber added a *social support/educational* frame and a *social/economic/political* frame to this scheme [19].

Other studies have identified frames based on how mass media discourse attributes responsibility for the causes and solutions of cancer [20]. Clarke and Everest divided cancer-related news into a *lifestyle* frame, which focuses on individual responsibility and solutions; a *political/economy* frame, which emphasizes the corresponding societal aspects; and a *medical* frame, which underscores biological explanations and biomedical solutions [21].

Another focus of research concerns the use of metaphors. Many studies have addressed the use of *war* metaphors and the depiction of cancer as an *enemy* [22]. Sontag also demonstrated how cancer often functions as a metaphor for both monstrosity and uncontrolled growth, whereas Sontag [3] and others have discussed a *mystical*, *alternative*, and *New Age* perspective to cancer. Finally, multiple studies have described how cancer is presented from a personal and psychological perspective, tending to describe the experience as a *journey*, *heroic struggle*, or *test of character* [23].

In summary, the literature on the framing of cancer discourse is rich and insightful but also quite fragmented and inaccessible to the general public. We find no study that combines the different frames, perspectives, and metaphors in an applicable educational tool for analyzing the contextual aspects of cancer discourse. In this study, therefore, we have developed a unified framing scheme, which facilitates critical reading of cancer-related information across a wide range of themes and context.

## Material and Methods

Drawing on the seminal work of Goffman [12], we regard frames as “schemata of interpretation” by which people make sense of issues and events. Further elaborated by Entman [22], these mental frames are embodied in the keywords, metaphors, concepts, symbols, and visual images represented in different items of communication.

Framing analysis is the process of identifying and exploring such frames and may be either a qualitative or quantitative method. For this study, we applied a qualitative approach, looking to characterize how the concept of cancer is framed from a newly selected material. The process was primarily inductive, but it was also informed by the literature outlined above. To validate, refine, and supplement previous research in the field, we set out to analyze a wide body of public discourse concerning cancer, and we found that a broad selection of online newspaper articles represented a pertinent source of information. Including news reports, interviews, features, editorials, commentaries, book reviews, and informational articles about health and science, this material conveyed many different societal voices and represented a comprehensive selection of cancer-related information. Moreover, online newspaper articles represented an easily definable material, readily available in searchable databases, also for scrutiny by other researchers.

To gain insight into the contemporary European context in two different languages, we chose to analyze leading daily newspapers from the UK and Norway in the period from 2013 to 2018. To increase representativeness of the study regarding the entire landscape of journalistic styles and the respective socioeconomic readerships, we included equal numbers of articles from an elite, a mid-market, and a tabloid newspaper from each country. Respectively, we selected *The Guardian* (GU), the *Daily Mail* (DM), and *The Sun* (SU) from the UK, and *Aftenposten* (AF), *Dagbladet* (DB), and *Verdens Gang* (VG) from Norway, building on the previous classification by Carver et al. [24].

Cancer-related articles were retrieved from the electronic databases Factiva (UK) and Atekst (Norway) by searching for *cancer* and *kreft*, respectively. To achieve a sample, both random and balanced, over the entire time period, the resulting lists of articles for each newspaper were sorted by date, and 100 articles per newspaper were selected at regular intervals. Irrelevant results referring to the cancer zodiac or passing mentions of the word *cancer*, for example, in the name of an organization, were excluded and replaced by the subsequent article in the list.

Using previously described frames and metaphors as a starting point, we then conducted a systematic framing analysis of the 600 articles. The three coders (N.P., C.M., J.B.) began analyzing identical sets of five articles, addressing the question: what kind of problem or issue is cancer according to this article? Recognizing that one article could comprise several cancer frames, we highlighted which text corresponded to which nascent frame, subsequently comparing and discussing coding until consensus.

After five such rounds of coding, a clear pattern started to emerge, and for the rest of the material, we only discussed articles that the coders identified as ambiguous or incompatible to our previous classifications. Aiming to develop an applicable educational tool, we sought a pragmatic balance between a framing scheme sensitive to the nuances of cancer discourse and one that was easy to explain and simple to use. Throughout the process, we also compared the emerging frames with trends and perspectives identified in previous research (presented above).

The study did not involve human subjects or sensitive information and required no ethical approval.

## Results

### Nine Cancer Frames

Integrating previous research and our own analysis of cancer-related newspaper articles, we identified nine distinctive cancer frames (Table 1). The frames were specifically classified by how they contextualize the concept of cancer, and we here present the characteristics of each frame and how it is differentiated from the other frames:

**Table 1:** Classification of cancer frames

Frame	Presenting cancer	Keywords
Biomedical	As a biological phenomenon, using scientific and medical terminology	Genetics, gene, mutations, anatomy, cells, tumor, organ, growth, chemo, medication, therapy
Epidemiologic	At the level of populations and public health in terms of statistics	Risk, survival, prognosis, incidence, screening
Environmental	As an environmentally caused phenomenon, related to exposure or lifestyle	Cause, prevent, smoking, alcohol, diet, pollution, radiation, toxic
Personal	As a personal and psychological issue focusing on the perspective of the individual, family, and friends	I, family, grief, pain, anxiety, loss, remorse
Sociopolitical	As a social or political issue, including cultural, educational, and ethical aspects	Equality, stigma, gender, race, prejudice, responsibility, campaign
Economic	As a financial issue, including health economics, fundraising, research funding, and business	Cost, financial, funding, industry, company
Alternative	In terms of New Age, anti-establishment, pseudoscience, supernatural, and serendipity	Energy, healing, power, vibrations, luck, paleo diet, herbs
Antagonist	As an adversary or challenge	Fight, beat, kill, win, lose, war, battle, enemy, intruder, aggressor
Symbolic	As a metaphor or simile for something very bad	Infectious, evil, invasive, spreading

#### The Biomedical Frame

An obvious and intuitive way of framing cancer is to present it as a disease: a biomedical problem characterized by the uncontrolled growth and spread of cells within the body, similar to how cancer is presented in a textbook of pathology. This frame was also prevalent in our material of newspaper articles. These articles focused on the physical and technical aspects of cancer and cancer treatment: “Its growth is driven by cancerous stem cells that are resistant to chemotherapy and radiation” (DM008). “Researchers have found the MC1R gene variant increases the number of mutations in skin cancer cells, multiplying the risk” (SU030).

Such articles typically described cancer at the level of cells, organs, or the body system, often in terms of genetic mutations and biological mechanisms. They depicted cancer as a biological phenomenon, a tumor or growth that might spread, invade, or metastasize and could also present information about symptoms, diagnostics, and therapeutic principles. The topic was often related to cancer research, and the related articles frequently cited biomedical researchers or medical professionals.

This frame is similar to the *medical* frame proposed by Clarke and Everest [21], which “depicted cancer as a physiologically based pathology explained and discussed within biomedicine.” The frame is characterized by reference to aspects like genes, cells, organs, medications etc. and is primarily delimited by the *epidemiological* and *environmental* frames (below). As elaborated by Clarke and Everest [21], the *biomedical* framing tends to treat cancer as a technical problem. It emphasizes treatment rather than prevention and is typically focused on solving the problem of cancer.

#### The Epidemiological Frame

Some articles presented cancer in terms of numbers and statistics, typically related to different groups and populations. Such accounts often concerned incidence and prevalence: “Around 10,300 cases of bladder cancer are diagnosed every year among the UK population” (DM083). There were also frequent presentations of prognosis: “Around 50 to 60 per cent are alive after three years and in the patients alive after five years there is a chance that the cancer will never return” (DM005). Some concerned cancer risk factors: “Men over 50 and men with a family history of prostate cancer also face a higher than average risk of the disease” (GU032). Others concerned screening and early detection: “early detection and treatment through cervical screening in the UK can prevent up to 75% of cervical cancers from developing” (GU001). Finally, there were articles that related cancer to the aging population: “Most cancers are a result of ageing as people are less likely to die from infectious diseases and advances in medical science are keeping more alive after heart attacks and strokes and with other medical problems” (GU083).

This frame is characterized by how it regards cancer in terms of numbers and distribution in populations. It bears some resemblance to the *basic information* frame of Andsager and Powers [18] but is more narrowly defined by its quantitative and statistical aspects. It is further delineated by the *biomedical* and the *environmental* frame. The *epidemiological* frame draws attention to the size of the cancer problem. It regards life and death in terms of numbers and is also unique in how it explains the cancer epidemic as a consequence of the aging population.

#### The Environmental Frame

Some articles could be characterized by how they attributed cancer to environmental factors, either related to lifestyle or more involuntary exposure to carcinogens: “The council wants sunbed salons to be regulated, to help halt the worrying rise of skin cancer” (DM088); “…a study which found an association between pesticide use and non-Hodgkin lymphoma” (GU006). Such articles describe behavior or exposure that promote or protect against cancer development, typically including factors like smoking, diet, sun-tanning, alcohol, and pesticides.

Clarke and Everest [21] have previously proposed a *lifestyle* and a *political/economy* frame, which both encompass elements of environmental causality. However, while these frames focus on individual and societal responsibility, we propose a unified *environmental* frame specifically defined as discourse that relates cancer to environmental causes. This frame encompasses inadvertent exposures to carcinogens as well as lifestyle-related factors like diet, smoking, and lack of exercise, regardless of responsibility. Discourse concerning aspects related to responsibility, blame, or shame of cancer is instead encompassed by the *personal* and the *sociopolitical* frame (below).

#### The Personal Frame

Many articles focused on personal and psychological aspects, regarding cancer from the perspective of individual patients and their families: “The family slowly began to adjust to life with Angie’s cancer hanging over them” (GU098), “‘Cancer never occurred to me,’ she recalls today, four years later. ‘I was 36, I had two young children and in the space of a few days my life had changed forever’” (DM086).

This way of framing cancer is similar to the *personal stories* frame of Andsager and Powers [18] and Park and Reber [19] and also encompasses frames and themes which present cancer as a personal fight, a test of character, a journey of growth, or simply as an arduous experience [23]. The *personal* frame is defined by discourse that describes cancer as a personal matter, often related to psychological distress and interpersonal relations. It regards cancer in terms of experience and emotions and contrasts the scientific and reductionist perspectives of the three above described frames.

#### The Sociopolitical Frame

Some of the newspaper articles framed cancer as a societal and/or political problem, for example, related to social disparities concerning age, gender, and socioeconomic status: “There are generational issues. Young women are quite comfortable about talking about this whereas the older generation aren’t” (DM058); “Boys are being denied protection against the risk of cancer because they are not routinely offered the same vaccination as girls” (GU082). Other articles concerned stigma and prejudice: “Twenty years ago people never mentioned the ‘c’ word” (GU074); “One patient I heard about told her friends she had breast cancer rather than lung cancer, because breast cancer was so much more acceptable and less judged” (GU067). There were accounts of the ethical problems of cancer: “Do the criteria of seriousness imply that people who get cancer at the age of 20 or 80 are assured the same treatment?” (AF046). There were examples of how cancer is affected by social interactions: “The researchers said a watchful husband or wife made it easier to catch the disease early” (DM089). There were overarching political aspects: “This all reflects a system that’s failing to meet the needs of people with cancer or suspected cancer” (GU019). And finally, there were accounts related to population health initiatives: “What can we do to make more black men understand the added danger they face and take the necessary action that could save their lives?” (GU023).

Combined, this way of framing cancer is characterized by its relations to aspects like social disparities, stigma, prejudice, social structures, social responsibility, and politics. It contains elements from the *political/economy* frame proposed by Clarke and Everest [21] and the *social support/educational* and *social/economic/political* frame of Park and Reber [19], and we recognize that there are several different ways to combine and subcategorize these different elements. However, aiming for simple definitions and distinct delimitations, we concluded on a combined *sociopolitical* frame, whereas all economic aspects were organized in a separate frame (below).

The macro perspective of the *sociopolitical* frame contrasts the individualized perspective of the *personal* frame. It regards cancer in a larger context and may as such be regarded as contrary to the reductionist perspective of the *biomedical* frame. The elevated point of view is somewhat similar to the *epidemiological* frame, but instead of presenting statistics, it focuses on the conflicts and dilemmas of cancer.

#### The Economic Frame

Many of the articles presented cancer as an economic issue. This frame was primarily related to cancer’s cost for either the patient or the public health system: “Nivomulab costs around 60,000 to 100,000 lb a year for a lung cancer patient” (DM022); “Many people with cancer will feel cold and lonely due to the disease’s financial impact” (SU044). Another economic perspective concerned charity and fundraising: “the fundraising director at the cancer charity Antony Nolan, said: ‘Hopefully we’ll raise over £600,000 and have over 255 runners’” (GU030). Other articles looked at cancer from the perspective of the pharmaceutical industry: “The firm also signed a clinical trial collaboration with the Japan’s Kyowa Hakko Kirin for a study that will assess combinations of the two companies’ cancer immunotherapy treatments” (GU072).

This *economic* frame views the cancer in terms of financial resources, fundraising, and business development. It is closely related to the *sociopolitical* frame (above) but at the same time easily distinguished by its pecuniary perspective. Moreover, a separately defined *economic* frame emphasizes the enormous economic implication of cancer [2].

#### The Alternative Frame

Some articles included perceptions which to varying degrees departed from established scientific understandings of cancer. Some gave alternative explanations for the cause of cancer: “If I was going to attribute my prostate cancer to anything, it would be that my body energy vibration, the balance of my body, was wrecked by what was going on” (DM032). Others made claims about alternative therapies: “[T]he spice [turmeric] may play a significant role in preventing or treating lung disease, brain disease and a variety of cancers – including multiple myeloma, colon cancer and pancreatic cancer” (DM038). There were also allusions to skepticism towards the mainstream medical and pharmaceutical industries: “I have news that Big Pharma doesn’t particularly want to hear. The ingredients for that pill are probably already right there on your kitchen shelves” (DM038).

Spanning from New Age philosophy to traditional religious considerations and more causal superstition, this way of framing cancer is defined by its metaphysical, mystical, or pseudoscientific perception of cancer. This *alternative* frame stands in contrast to the scientifically based discourse of the *biomedical*, *epidemiological*, and *environmental* frame. It has a strong position in public discourse and poses a challenge to efforts to promote evidence-based understanding of cancer [3].

#### The Antagonistic Frame

Consistent with previous research, we found ample use of conflict metaphors in the material of newspaper articles. Such accounts often concerned individual cancer patients: “Sir Michael Parkinson is winning his battle with prostate cancer” (SU092); “I don’t plan to give up without a fight” (SU039). Some also deliberated on the matter: “Marshall dislikes the ‘battle with cancer’ narrative, and is loathe to describe her relationship with it as such. ‘I know it’s a cliché, but I would rather die standing than live on my knees’” (GU064). Other articles presented cancer as a fight within the body, often in combination with the biomedical frame: “Her team is now hoping that it can identify how cancer hijacks the body’s cells, and develop treatments which would destruct cancer cells without harming healthy cells” (SU057); “immunotherapy […] works by ‘switching on’ the body’s immune system to fight cancer cells” (DM005). Finally, there were articles that depicted cancer as a fight at the societal and political level: “It is up to us in the community to act […] Ignoring cancer won’t beat it” (GU023).

Overall, we found that cancer was framed as an adversary in several different contexts, often in combination with other frames (elaborated below). This kind of framing has been criticized for promoting unrealistic expectations, disempowering patients, and undermining preventative behaviors [22]. Concurrently, the *antagonistic* frame is a powerful tool for rallying support and sympathy, at the individual as well as the political level, and there are valid arguments for framing cancer as an enemy, also from a biological perspective. This frame has a prominent position in public cancer discourse, and its conflicting function calls for special attention.

#### The Symbolic Frame

Some articles used the concept of cancer as a symbol to describe something else. Typically, they applied cancer in metaphors or similes to describe other entities or phenomena as evil, invasive, or spreading: “Corruption can no longer be described as a cancer on the system: it is the system” (DM034); “this new censorship is spreading like a cancer across British universities” (DM045).

This *symbolic* frame uses the concept of cancer to describe something else and is thus different from discourse that uses metaphors to describe cancer [3]. It is also categorically different from all the other frames since the discourse is not actually about cancer. Nevertheless, we concluded that the *symbolic* frame represents an aspect that both reflects and influences people’s perception of cancer. It may reinforce the negative connotations of the disease and contribute to further stigmatization of patients. Although it is not a framing of cancer per se, we thus argue that the *symbolic* frame belongs in a comprehensive scheme for analyzing cancer discourse.

### Frame Combinations

Whereas framing analysis often aims to identify a dominant frame for each article, the above described framing scheme allows for a more detailed analysis. Many of the articles in the material included more than one cancer frame, and the analysis revealed how frames combined to produce more complex messages.

Some frames combined to evoke a new composite meaning. For instance, the *antagonistic* frame combined with the *biomedical* frame to present cancer as a tangible enemy, which is fought with biotechnology: “[I]mmunotherapy […] works by ‘switching on’ the body’s immune system to fight cancer cells” (DM005). In other cases, the *antagonistic* frame combined with the *sociopolitical* and *economic* frame to present cancer as a public enemy. This combination typically reflected the *war on cancer* mentality, often aiming for *a cure* and often appearing in articles calling on people to promote research funding and donate to cancer charities: “[W]ith YOUR help, you could help us smash our £1million target […] helping scientists make positive steps towards a cure” (SU028). Similarly, the *antagonistic* frame combined with the *personal* frame to present the psychological hardship of cancer: “[H]e had ‘fought the constant recurrences of his cancer with dogged courage’” (GU095).

Another combinatory modification was seen for the alternative frame. Combining the *alternative* frame with the *personal* frame typically presented cancer as a spiritual and metaphysical phenomenon: “[H]e’d ask a child if their cancer was caused by negative energy” (DM032). Conversely, the *alternative* frame combined with the *biomedical* and *environmental* frame to compose a pseudoscientific message: “Ounce for ounce, herbs and spices have more antioxidants than any other food group. This means they can help prevent the initial triggering of mutations in your DNA that could lead to cancer or other diseases” (DM046). These combined messages have varying degrees of scientific validity. They may be difficult to recognize and assess, without expert knowledge, and deciphering and countering such pseudoscientific discourses represent a key challenge in cancer communication.

**Fig. 1:**
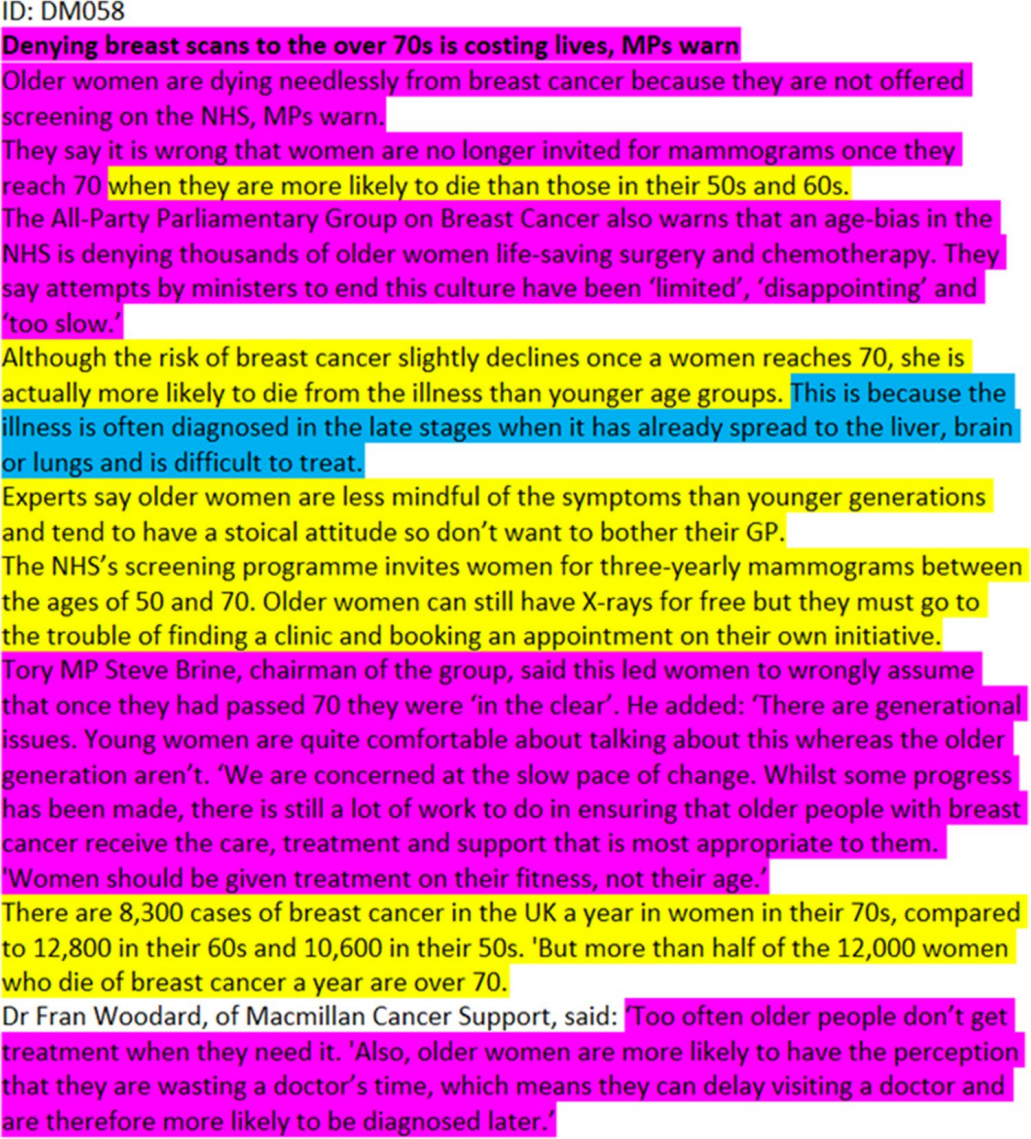
This article combines the thematically related *sociopolitical* frame (magenta) and *epidemiological* frame (yellow) to present a public health perspective. Notice also a limited account of the *biomedical* frame (blue), which complements the public health perspective with information about the underlying biology and pathology of cancer

**Fig. 2:**
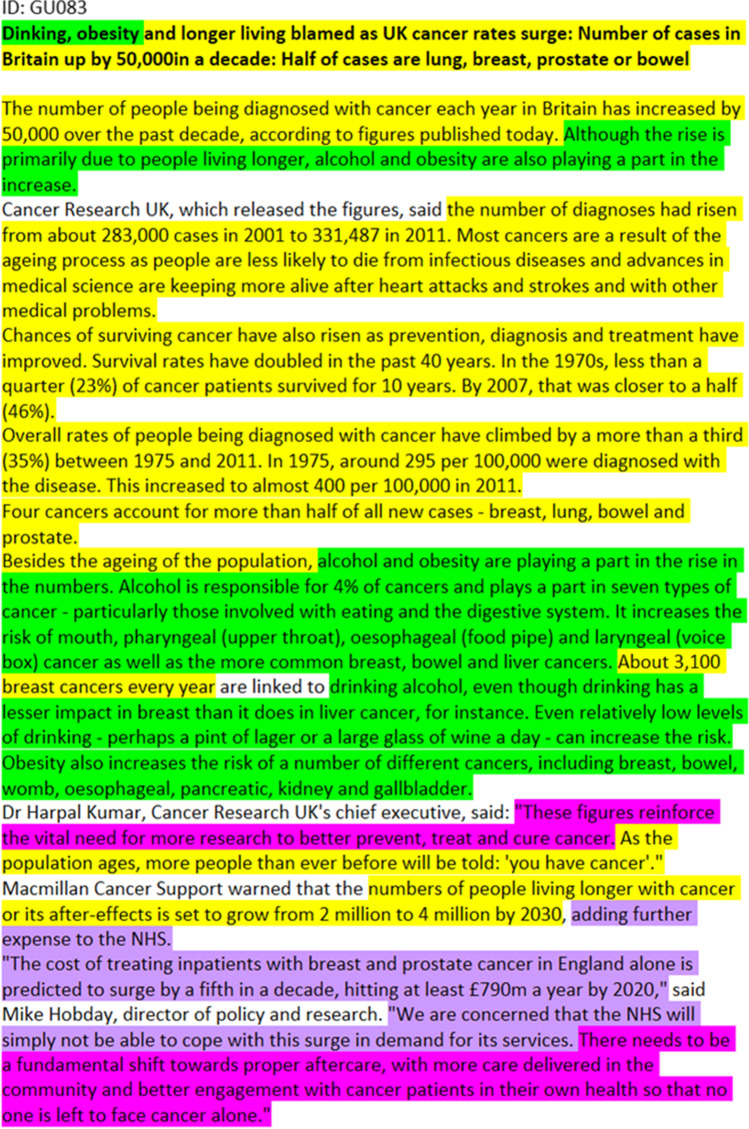
This article primarily combines the *epidemiological* frame (yellow) and the *environmental* frame (green) to form a prevention-oriented public health perspective. Towards the end, it then switches to the *sociopolitical* (magenta) and *economic* (violet) frame, appealing for more cancer research, emphasizing the growing cost of cancer for society and the need for change in the healthcare system

Some frame combinations did not modify meaning but were associated because of thematic relationship. The *epidemiological*, *sociopolitical*, and *economic* frames all presented the issue of cancer at the level of population and society, and their permutations tended to converge to a public health perspective. In one article, for example, the *epidemiological* frame was used to demonstrate the quantitative scope of cancer, and the *economic* frame related those numbers to financial implications, while the *sociopolitical* frame used the unfavorable scenario as grounds to campaign for improvements of care (Fig. 1). Similarly, the *environmental* frame was often accompanied by *epidemiological* information, typically to substantiate links between exposure and cancer risk (Fig. 2).

Other frames were inherently distinct but complemented one another to produce synergic effects. For example, predominantly biomedically framed articles sometimes included elements of the *personal* frame, making scientific information more relatable: “Radiotherapy can be effective, but there is a risk of damaging healthy tissue. However, a highly-targeted treatment can zap the tumour in just five days, leaving surrounding organs intact. Dr John Sheehy, 70, a scientist from Marlow, Buckinghamshire, underwent the therapy” (DM090). Conversely, elements of the *biomedical* frame added authority and factual substance to personal stories about cancer: “If I’d hoped my cancer was early stage and non-invasive, my follow-up appointment that Friday revealed a different story […] It was likely to be grade two or three, depending on whether he discovered a spread to my lymph nodes during my mastectomy” (DM056).

## Discussion

In this study, we have developed an applicable tool for analyzing cancer communication. Specifically, we have identified and categorized nine different cancer frames, which may be recognized in cancer discourse across different themes and context. This unified framing scheme may be used in education and further research to facilitate cancer literacy.

The analyzed material of newspaper articles was limited to the recent 5-year period and a Western European context, and there may be historical or cultural aspects to cancer that are missing. Moreover, this qualitative, largely inductive study aimed to identify and characterize a set of frames based on obtaining consensus between the coders and does not include an assessment of inter-coder reliability. A deductive analysis, for example, to explore differences between countries or media sources, will require a quantitative design and is deferred to future studies.

We also acknowledge that our framing scheme is merely one of several possible ways to classify public cancer discourse and that different framing schemes address different problems of communication. Yet, for the purpose of developing a simple framework that may be used for educational purposes, to analyze cancer discourse across themes and topics, we believe that this framing scheme represents a sensible balance between simplicity and discriminatory power.

Whereas the nine frames are related to previously described frames, there are also important differences. The *antagonistic* frame is clearly related to the vast literature (here represented by [3, 22]) which has analyzed and discussed the use of war metaphors in cancer discourse. Concurrently, however, we identified a clear distinction between this frame and a *symbolic* frame, which applies the word cancer as a metaphor or simile. Whereas the *antagonistic* frame presents cancer as an enemy, the *symbolic* frame uses the concept of cancer to characterize something else (e.g., corruption or Islamism) as a malicious foe.

We also identified an *environmental* frame, clearly defined by how it attributes cancer to exposure and lifestyle-related factors, and an *epidemiological* frame, which specifically concerns the statistical and population-based aspects of the disease. These frames are further defined by their distinction from the *biomedical* frame, which concerns the biological, diagnostic, and therapeutic aspects of cancer.

Another novel feature of the presented framing scheme is the ability to identify and explore a variety of frame combinations. The use of one frame may be modified, reinforced, or complemented by other frames, and an article may represent different permutations of the nine frames. The framing scheme may thus be used to dissect composite framing effects in more complex messages. Such analysis may reveal the underlying strategies or conflicts in cancer communication, which may be difficult to identify without a comprehensive framing scheme.

Combined, the nine cancer frames may be used as a tool for cancer education. The concept of framing, and how it affects communication, is quite easy to understand and may be applied to different levels of education, from secondary school to PhD courses. As demonstrated by Carver et al. [25], a set of clearly defined frames was used as an educational tool that enabled students to analyze and explore genetic discourse in a systematic manner, which prompted scientific understanding as well as media literacy. Similarly, the above presented framing scheme is especially relevant for an interdisciplinary educational approach, combining a biomedical and a societal perspective to cancer.

Groups of students may be presented with the framing scheme and asked to identify which frames are used in different media articles [25]. Each frame may be assigned a specific color, as demonstrated in Figs. 1 and 2, and the students are provided highlighter pens to mark the corresponding sections of the text. They are then asked to compare and discuss their individual assessments, thereby facilitating a systematic and critical analysis of cancer communication. Using this framing scheme as an educational tool may thus increase awareness and promote critical thinking and reading skills, which are increasingly important for individual as well as political decision-making with regard to cancer [6].

Moreover, the framing scheme may be used by professionals, including journalist, researchers, and healthcare providers to analyze their own as well as others’ cancer-related communication. This ability to critically assess cancer discourse is of special relevance to healthcare professionals, who communicate about cancer on a regular basis. How they frame the concept of cancer may have profound implications, not only for the patient’s understanding of disease, but may also influence their preventive behavior and therapeutic compliance [7]. More attention to cancer literacy among patients as well as healthcare providers may thus have a positive impact on a range of decisions and actions in the cancer continuum [8, 9].

With regard to further research, a unified framing scheme may be applied in quantitative analyses to reveal underlying patterns in communication [24]. The cancer frames may be used to uncover differences in cancer communication between countries and media sources, over time, or with regard to different types of cancer. We also anticipate qualitative and quantitative studies to explore the prevalence of these frames in different types of cancer-related information, from news reports to patient blogs and information leaflets.

In conclusion, we have developed a practical tool for contextual analysis of cancer-related information. The nine cancer frames are based on a synthesis of previous research as well as an independent analysis of a large, international material of newspaper articles. This framing scheme may be applied in further research and educational initiatives to promote cancer literacy and a better understanding of cancer as a multi-dimensional problem in science and society.
